# Platelet BACE1 levels as a possible biomarker for Alzheimer’s disease

**DOI:** 10.1186/1750-1326-8-S1-P14

**Published:** 2013-09-13

**Authors:** Boris Decourt, Aaron Walker, Amanda Gonzales, Michael Malek-Ahmadi, Carolyn Liesback, Kathryn Davis, Christine Belden, Sandra Jacobson, Marwan Sabbagh

**Affiliations:** 1 Banner Sun Health Research Institute, Sun City, AZ, United States Minor Outlying Islands

## Background

To date there is no validated peripheral biomarker to assist with the clinical diagnosis of Alzheimer’s disease (AD). On the other hand, platelet proteins have been studied as AD biomarkers with relative success. In the present study we investigated whether platelet BACE1 levels differ between AD and cognitively normal (CN) control patients.

## Materials and methods

We collected blood samples from 12 CN and 15 AD subjects using a standardized procedure. All samples were processed within 5-60 min after collection. Blood fractions were separated by successive centrifugations: red blood cells, platelet-rich fraction, and platelet-poor plasma. Both platelet and plasma samples were analyzed using a newly developed BACE1 ELISA method and Western blotting.

## Results

ELISA analysis showed that platelet BACE1 levels were significantly lower in AD compare to CN subjects (12% decrease, p<0.05). These data were supported by Western blotting which indicated that several BACE1 isoforms (37, 45, 56, 70 kDa) were significantly less abundant in AD platelets. However, no significant difference between the two groups was noted in plasma fractions.

## Conclusions

Our pilot study suggests that circulating platelets possess less BACE1 protein AD subjects. This provides evidence for testing platelet BACE1 levels as a peripheral AD biomarker using our novel, sensitive and inexpensive ELISA method. We are currently investigating this hypothesis using larger groups as well as Parkinson’s disease subjects to verify the specificity of the approach.

